# Some Metabolites Act as Second Messengers in Yeast Chronological Aging

**DOI:** 10.3390/ijms19030860

**Published:** 2018-03-15

**Authors:** Karamat Mohammad, Paméla Dakik, Younes Medkour, Mélissa McAuley, Darya Mitrofanova, Vladimir I. Titorenko

**Affiliations:** Department of Biology, Concordia University, 7141 Sherbrooke Street, West, SP Building, Room 501-13, Montreal, QC H4B 1R6, Canada; karamat.mohammad@concordia.ca (K.M.); pameladakik@gmail.com (P.D.); writetoyounes@gmail.com (Y.M.); melissa.mcauley@concordia.ca (M.M.); mitrofanova_darya@hotmail.com (D.M.)

**Keywords:** yeast, chronological aging, mechanisms of longevity regulation, metabolism, cell signaling, second messengers, mitochondria, interorganellar communications, proteostasis, regulated cell death

## Abstract

The concentrations of some key metabolic intermediates play essential roles in regulating the longevity of the chronologically aging yeast *Saccharomyces cerevisiae*. These key metabolites are detected by certain ligand-specific protein sensors that respond to concentration changes of the key metabolites by altering the efficiencies of longevity-defining cellular processes. The concentrations of the key metabolites that affect yeast chronological aging are controlled spatially and temporally. Here, we analyze mechanisms through which the spatiotemporal dynamics of changes in the concentrations of the key metabolites influence yeast chronological lifespan. Our analysis indicates that a distinct set of metabolites can act as second messengers that define the pace of yeast chronological aging. Molecules that can operate both as intermediates of yeast metabolism and as second messengers of yeast chronological aging include reduced nicotinamide adenine dinucleotide phosphate (NADPH), glycerol, trehalose, hydrogen peroxide, amino acids, sphingolipids, spermidine, hydrogen sulfide, acetic acid, ethanol, free fatty acids, and diacylglycerol. We discuss several properties that these second messengers of yeast chronological aging have in common with second messengers of signal transduction. We outline how these second messengers of yeast chronological aging elicit changes in cell functionality and viability in response to changes in the nutrient, energy, stress, and proliferation status of the cell.

## 1. Introduction

Studies of the budding yeast *Saccharomyces cerevisiae* have led to the discovery of genes, signaling pathways, and small molecules that define the rate of cellular aging in this unicellular eukaryote [1,2,3]. Some of the genes, signaling pathways, and small molecules discovered in *S. cerevisiae* have been later found to play essential roles in cellular and organismal aging in diverse species of multicellular eukaryotes; it is believed, therefore, that the mechanisms underlying aging and longevity assurance have been conserved throughout the course of evolution [4,5,6]. Thus, the use of *S. cerevisiae* as a model organism is fundamental for the progress in aging research [2,3,4].

There are two different ways of studying aging in *S. cerevisiae*; each of these ways investigates a different mode of yeast aging.

A so-called replicative mode of yeast aging is monitored by counting the total number of asymmetric mitotic divisions—each producing a small daughter cell—that a mother cell could undergo on the surface of a solid nutrient-rich medium before it becomes senescent [2,7,8]. Yeast replicative aging has long been considered to resemble the aging of those cells in humans and other mammals that are able to divide mitotically; among these mitotically active cells are lymphocytes, monocytes, granulocytes, fibroblasts, and some stem cell types [2,4,6,7,8]. Recent studies have revealed that (1) many genes that modulate aging of post-mitotic cells in adults of the nematode *Caenorhabditis elegans* also influence yeast replicative aging [9,10,11]; and (2) many hallmarks of aging characteristic of post-mitotic cells in humans and other mammals are also cellular hallmarks of yeast replicative aging [12]. Hence, it is conceivable that the replicative mode of yeast aging may also mirror the aging of post-mitotic cells and even the aging of the entire organism in nematodes, humans, and other mammals [9,10,11,12].

A so-called chronological mode of yeast aging is monitored by determining how long a yeast cell cultured in a liquid medium can retain viability after it undergoes cell cycle arrest and enters a state of quiescence [2,13,14]. Yeast chronological aging is believed to model the aging of human and mammalian cells that lose the ability to divide mitotically; these post-mitotic cells include adipocytes, mature muscle cells, and mature neurons [2,14,15]. The chronological mode of yeast aging is also considered to be a simple model of organismal aging in multicellular eukaryotes [14,16].

Although the replicative and chronological modes of aging in yeast are usually examined separately from each other, recent evidence indicates that these two modes of yeast aging most likely converge into a single aging process [17,18,19].

Here, we review mechanisms through which the spatiotemporal dynamics of changes in the concentrations of some metabolites regulate the longevity of chronologically aging yeast. Based on the important advance in our understanding of these mechanisms, we conclude that a distinct group of metabolites act as second messengers that define the pace of yeast chronological aging.

## 2. Concentrations of Some Metabolites Define the Rate of Chronological Aging in Yeast

Recent studies have demonstrated that the intracellular and extracellular concentrations of some key metabolites play essential roles in regulating the longevity of chronologically aging *S. cerevisiae* [2,3,4,5,15,16,20,21,22,23,24,25,26,27,28,29,30,31,32]. These key metabolites are detected by certain protein sensors, which respond to concentration changes of the metabolites by altering the efficiencies of cellular processes known to define yeast chronological lifespan (CLS) [2,3,4,16,20,21,22,23,24,25,26,27,28,29,30,31,32]. In this section, we describe the metabolites whose concentration changes affect the pace of yeast chronological aging and discuss mechanisms through which these key metabolites influence yeast CLS.

### 2.1. NADPH

**Figure 1:**
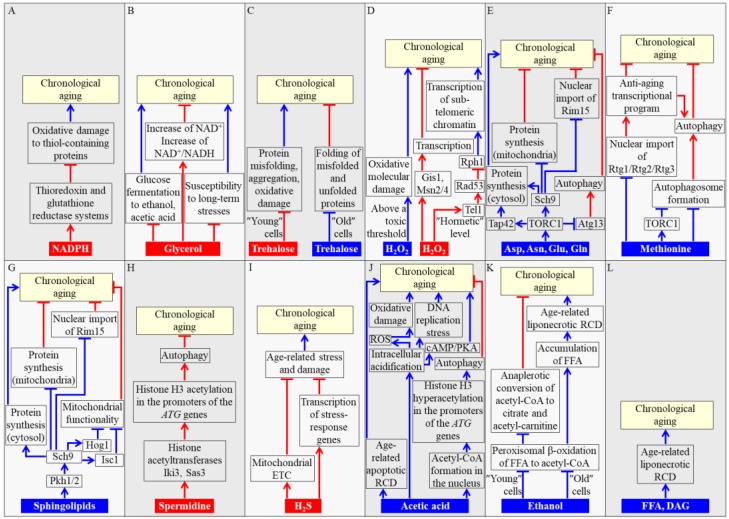
Mechanisms through which the concentrations of some key metabolites define the rate of chronological aging in the yeast *Saccharomyces cerevisiae*. These key metabolites include NADPH (**A**), glycerol (**B**), trehalose (**C**), H_2_O_2_ (**D**), the amino acids aspartate, asparagine, glutamate and glutamine (**E**), methionine (**F**), sphingolipids (**G**), spermidine (**H**), H_2_S (**I**), acetic acid (**J**), ethanol (**K**), as well as FFA and DAG (**L**). These key metabolites are detected by a distinct set of ligand-specific protein sensors that respond to the concentration changes of the metabolites by altering the rates and efficiencies of longevity-defining cellular processes, thus creating a pro- or anti-aging cellular pattern and affecting the pace of yeast chronological aging. See text for more details. Activation arrows and inhibition bars denote pro-aging processes (displayed in blue color) or anti-aging processes (displayed in red color). Pro-aging or anti-aging metabolites are displayed in blue color or red color, respectively. Abbreviations: Asp, aspartate; Asn, asparagine; *ATG*, autophagy-related genes; ETC, electron transport chain; FFA, free (non-esterified) fatty acids; DAG, diacylglycerol; Glu, glutamate; Gln, glutamine; PKA, protein kinase A; RCD, regulated cell death; ROS, reactive oxygen species.

NADPH is generated in the Zwf1- and Gnd1-dependent reactions of the pentose phosphate pathway operating in the cytosol of *S. cerevisiae*, as well as in the Ald4-, Pos5-, Mae1-, and Idp1-driven reactions confined to yeast mitochondria [3,20,33]. NADPH is indispensable for the growth and viability of yeast cells because it serves as the major source of reducing equivalents for amino acid, fatty acid, and sterol synthesis [20,33]. In addition, NADPH has a specific role in longevity assurance of chronologically aging yeast because it provides electrons for thioredoxin and glutathione reductase systems (TRR and GTR, respectively) [34,35]. Both these NADPH-dependent reductase systems play essential roles in the maintenance of intracellular redox homeostasis, thereby decreasing the extent of oxidative damage to thiol-containing proteins that reside in the cytosol, nucleus, and mitochondria of yeast cells [34,35]. Such NADPH-driven, TRR- and GTR-dependent protection of many thiol-containing proteins from oxidative damage slows down yeast chronological aging (Figure 1A) [35].

### 2.2. Glycerol

Glycerol is one of the products of glucose fermentation in the cytosol of *S. cerevisiae* cells [33]. An increase in the intracellular and extracellular concentrations of glycerol has been shown to decelerate yeast chronological aging [15,36]. Three mechanisms have been proposed to underlie such aging-delaying action of glycerol. These mechanisms are depicted in Figure 1B and outlined below. First mechanism: an increase in glucose fermentation to glycerol lowers metabolite flow into glucose fermentation to ethanol and acetic acid, both of which accelerate yeast chronological aging (Figure 1B) [3,15,36,37]. Second mechanism: glycerol decreases the susceptibility of yeast cells to long-term oxidative, thermal, and osmotic stresses; an age-related intensification of all these stresses is a potent pro-aging factor in chronologically aging yeast (Figure 1B) [3,36]. It is presently unknown if this second mechanism involves some protein sensors that respond to an increase in glycerol concentration by stimulating certain stress response processes in yeast cells. Third mechanism: an increase in glucose fermentation to glycerol allows an increase in both the intracellular concentration of NAD^+^ and the intracellular NAD^+^/NADH ratio, thereby setting up a pro-longevity cellular pattern in chronologically aging *S. cerevisiae* (Figure 1B) [3,36].

### 2.3. Trehalose

Trehalose, a non-reducing disaccharide synthesized from glucose, has long been considered only as a reserve carbohydrate in *S. cerevisiae* cells [38]. However, recent evidence indicates that trehalose is also essential for regulating the longevity of chronologically aging yeast [37,39,40,41,42,43,44,45]. Depending on the chronological age of *S. cerevisiae*, trehalose exhibits either an anti-aging or pro-aging effect. Both these effects are due to the ability of trehalose to directly bind unfolded domains of proteins, thereby modulating various aspects of cellular proteostasis throughout CLS. In chronologically “young” yeast cells that proliferate, trehalose assists in sustaining an anti-aging pattern of cellular proteostasis. This is because trehalose binding to newly synthesized proteins in these cells helps to decrease the misfolding, aggregation, and oxidative damage of such proteins (Figure 1C) [37,41,46,47]. In chronologically “old” yeast cells that enter a non-proliferative state, trehalose contributes to the establishment of a pro-aging pattern of cellular proteostasis. This is because trehalose binding to hydrophobic amino acid side chains of misfolded, partially folded, and unfolded proteins in these cells shields extended patches of hydrophobic amino acid residues from the molecular chaperones needed for the folding of such proteins (Figure 1C) [37,41,46].

### 2.4. Hydrogen Peroxide (H_2_O_2_)

H_2_O_2_ is the major molecule of reactive oxygen species (ROS) [48]. In chronologically aging yeast, H_2_O_2_ and other ROS are initially generated in mitochondria and peroxisomes as by-products of mitochondrial respiration and peroxisomal oxidative metabolism, respectively [21,22,23]. After being made in mitochondria and peroxisomes, H_2_O_2_ is released from these two organelles and may play a dual role in regulating yeast CLS [21,22,23]. If the intracellular concentration of H_2_O_2_ exceeds a toxic threshold, it accelerates the process of chronological aging by eliciting oxidative damage to proteins, lipids, and nucleic acids in various locations within the yeast cell [21,48]. If the intracellular concentration of H_2_O_2_ is maintained at a “hormetic” level (i.e., a sub-lethal concentration of H_2_O_2_ that is insufficient to damage cellular macromolecules), this ROS can activate at least two signaling networks that extend yeast CLS by creating an anti-aging cellular pattern [2,3,27]. One of these hormetic H_2_O_2_-responsive signaling networks activates the Gis1, Msn2, and Msn4 transcription factors; after being activated, Gis1, Msn2, and Msn4 establish an anti-aging cellular pattern by stimulating the transcription of many genes involved in carbohydrate and lipid metabolism, nutrient sensing, cell cycle progression, autophagy, stress response and protection, and stationary phase survival (Figure 1D) [2,49,50]. Another hormetic H_2_O_2_-responsive signaling network is initiated when the DNA damage response (DDR) kinase Tel1 phosphorylates and activates the DDR kinase Rad53; Rad53 then phosphorylates and inactivates the histone demethylase Rph1, thus creating an anti-aging cellular pattern by suppressing the Rph1-driven transcription of sub-telomeric chromatin regions (Figure 1D) [51,52]. Mechanisms though which the Rad53-dependent suppression of sub-telomeric transcription prolongs yeast CLS remain unknown [51].

### 2.5. Amino Acids

The synthesis of the amino acids aspartate, asparagine, glutamate, and glutamine from tricarboxylic acid (TCA) cycle intermediates occurs in mitochondria of yeast cells [20,33]. These amino acids then exit mitochondria and activate protein kinase activity of the target of rapamycin complex 1 (TORC1), a pro-aging regulator confined to the surface of yeast vacuoles (Figure 1E) [53,54,55,56]. Active TORC1 orchestrates the establishment and maintenance of a pro-aging cellular pattern by phosphorylating at least three downstream protein targets and altering their activities. As outlined below, each of these downstream protein targets of TORC1 defines yeast CLS because it regulates one or more longevity-defining cellular processes.

One of the proteins phosphorylated by TORC1 is the nutrient-sensing protein kinase Sch9. Once phosphorylated by TORC1, Sch9 influences some longevity-defining cellular processes as follows: (1) it promotes ribosome assembly and stimulates translation initiation, thus activating the pro-aging process of protein synthesis in the cytosol [57,58,59,60]; (2) it slows down the anti-aging process of protein synthesis in mitochondria [55,56,61,62]; and (3) it inhibits nuclear import of Rim15, a nutrient-sensing protein kinase that elicits an anti-aging cellular pattern by stimulating the Gis1, Msn2, and Msn4 transcription factors in the nucleus [63,64,65]; this pro-aging effect of Sch9 is reinforced by the nutrient-sensing protein kinase A (PKA), which impedes nuclear import of Msn2 and Msn4 (Figure 1E) [65,66,67].

Another downstream phosphorylation target of active TORC1 is Tap42, a protein that (akin to Sch9) stimulates the pro-aging process of protein synthesis in the cytosol by promoting ribosome assembly and activating translation initiation (Figure 1E) [58,68].

The autophagy-initiating protein Atg13 is also a downstream phosphorylation target of active TORC1 [69,70]. The TORC1-driven phosphorylation of Atg13 suppresses the anti-aging process of autophagy by inhibiting autophagosome formation in the cytosol (Figure 1E) [71,72,73]. Atg13 can also be phosphorylated by PKA, which strengthens the pro-aging effect of TORC1 by further suppressing the formation of autophagosomes [69,70,71,72,73].

Like aspartate, asparagine, glutamate, and glutamine, the amino acid methionine is a pro-aging metabolite in yeast that ages chronologically (Figure 1F). A likely pro-aging effect of methionine consists in stimulating the methylation of tRNAs in the cytosol [74]. The resulting decline in the concentration of non-methylated tRNAs weakens nuclear import of the Rtg1/Rtg2/Rtg3 heterotrimeric transcription factor needed for driving an anti-aging transcriptional program (Figure 1F) [74]. Another pro-aging effect of methionine is manifested in its ability to inhibit autophagy by activating TORC1 and/or by suppressing autophagosome formation in a TORC1-independent manner (Figure 1F) [75]. This shortens yeast CLS by decreasing the acidity of the vacuole and by eliciting the accumulation of extracellular acetic acid, a potent pro-aging metabolite [74,75,76,77].

### 2.6. Sphingolipids

Sphingolipids are pro-aging metabolites in chronologically aging yeast because some genetic and pharmaceutical interventions that change the intracellular concentrations of certain sphingolipids have been shown to prolong yeast CLS [24,28,29,32]. Mechanisms underlying the essential role of sphingolipids in defining yeast CLS have begun to emerge; these mechanisms are outlined below.

After being synthesized in the endoplasmic reticulum (ER), the sphingolipid backbone base phytosphingosine stimulates protein kinase activities of Pkh1 and Pkh2 (Pkb-activating kinase homolog proteins 1 and 2) [78,79]. Pkh1 and Pkh2 then phosphorylate and activate the nutrient-sensing protein kinase Sch9 [24,80,81]. Such Pkh1/2-dependent phosphorylation of Sch9 establishes a Pkh1/2-Sch9 branch of a network that integrates nutrient and sphingolipid signaling (Figure 1G) [24,29,32,81]. As mentioned in Section 2.5, the phosphorylated and therefore active form of Sch9 in this signaling branch accelerates yeast chronological aging by stimulating the pro-aging process of protein synthesis in the cytosol [57,58,59,60], decelerating the anti-aging process of protein synthesis in mitochondria [55,56,61,62] and inhibiting nuclear import of the nutrient-sensing protein kinase Rim15 (Figure 1G) [63,64,65]. The Pkh1/2-dependent phosphorylation of Sch9 also elicits the Sch9-driven phosphorylation and activation of: (1) Hog1, a mitogen-activated protein kinase that attenuates mitochondrial functionality; and (2) Isc1, an inositol phosphosphingolipid phospholipase C that is involved in the formation of ceramides from complex sphingolipids in yeast mitochondria (Figure 1G) [24,28,29,32]. This ability of the Pkh1/2-Sch9 signaling branch to promote the phosphorylation of Hog1 and Isc1 allows it to coordinate mitochondrial functionality and sphingolipid metabolism, thus making an essential contribution to longevity regulation in chronologically aging yeast [24,28,29,32].

### 2.7. Spermidine

The natural polyamine spermidine is an aging-delaying metabolite that extends yeast CLS [82,83,84]. In yeast cells, spermidine is synthesized from the amino acids arginine and methionine in a series of reactions confined to mitochondria, the cytosol, and peroxisomes [84]. Spermidine delays yeast chronological aging because it inhibits the histone acetyltransferases Iki3 and Sas3 (Figure 1H) [82]. Although such spermidine-dependent inhibition of the two acetyltransferases attenuates transcription of many nuclear genes because it impairs histone H3 acetylation at their promoter regions, the extent of histone H3 acetylation at the promoter regions of several autophagy-related (*ATG*) genes is increased in yeast cells that exhibit high spermidine concentrations [82]. This selectively activates the transcription of the *ATG* genes, thus enhancing the cytoprotective process of autophagy and delaying yeast chronological aging (Figure 1H) [82,83].

### 2.8. Hydrogen Sulfide (H_2_S)

H_2_S is a metabolite that plays an essential role in the delay of yeast chronological aging by caloric restriction (CR) [85], a dietary regimen that delays aging, increases lifespan, and improves healthspan in evolutionarily distant eukaryotes [1,86,87]. In yeast, this water- and fat-soluble gas can be generated endogenously via two different metabolic pathways. One pathway of H_2_S synthesis involves a unique yeast assimilation of exogenous inorganic sulfate [88]. Another pathway of H_2_S synthesis is an evolutionarily conserved trans-sulfuration pathway (TSP) of transfer from methionine to cysteine [88]. In yeast cultured in a liquid synthetic medium, only H_2_S that is endogenously synthesized via the TSP pathway and then released to the culture medium is responsible for yeast CLS extension under CR conditions [85]. Mechanisms through which an exogenous (extracellular) pool of H_2_S delays chronological aging of yeast limited in calorie supply remain to be determined. It has been suggested that low, hormetic concentrations of H_2_S may protect chronologically aging yeast from age-related stress and damage by accelerating the electron transport chain in mitochondria and/or by activating the transcription of many stress-response genes in the nucleus (Figure 1I) [85,88,89].

### 2.9. Acetic Acid

Acetic acid is one of the products of glucose fermentation by yeast cultured in a liquid medium under high oxygenation [2,15,33]. It is formed in the Ald6-dependent reaction that occurs in the cytosol and in the Ald4-driven reaction confined to mitochondria [3]. Acetic acid is a pro-aging metabolite that shortens yeast CLS [15,90].

Three mechanisms through which extracellular and/or intracellular pools of acetic acid may accelerate yeast chronological aging have been suggested, all based on known effects of these acetic acid pools. These mechanisms are outlined below.

First mechanism: acetic acid (and/or the acidification of the liquid culture medium it causes) may, directly or indirectly, elicit an age-related apoptotic mode of regulated cell death (Figure 1J) [15,90,91,92,93,94].

Second mechanism: extracellular acidification caused by the buildup of acetic acid in the culture medium may trigger intracellular acidification, which may then accelerate chronological aging by: (1) stimulating the pro-aging cyclic adenosine monophosphate (cAMP)/PKA signaling pathway; (2) increasing the concentration of ROS, thus eliciting oxidative damage to cellular macromolecules; and/or (3) enhancing DNA replication stress (Figure 1J) [2,90,92].

Third mechanism: nuclear transport of acetic acid and its subsequent Acs2-dependent conversion into acetyl-Coenzyme A (CoA) may suppress the transcription of nuclear *ATG* genes by causing histone H3 hyperacetylation at their promoter regions, thus slowing down the cytoprotective process of autophagy and accelerating yeast chronological aging (Figure 1J) [25,26,95,96].

It needs to be emphasized that exogenous acetic acid accelerates yeast chronological aging not only because it creates an acidic extracellular environment; indeed, neither other organic acids present in culture medium of aged yeast nor hydrochloric acid can shorten yeast CLS if added exogenously [15]. Moreover, only exogenous acetic acid at acidic pH, but not its conjugate base at neutral pH, exhibits the aging-accelerating effect in yeast [15]. It is conceivable, therefore, that only a combinatory action of acetic acid and an acidic extracellular environment is responsible for the acceleration of yeast chronological aging via the above mechanisms [15].

### 2.10. Ethanol

Yeast cells cultured in a liquid medium under low oxygenation produce ethanol as the main product of glucose fermentation in the Adh1-dependent reaction confined to the cytosol [2,3,13,14,33]. Ethanol is a pro-aging metabolite that shortens yeast CLS [37,97]. This pro-aging action of ethanol can be enhanced by the sirtuin deacetylase Sir2, which inhibits the Adh2-driven conversion of ethanol to acetaldehyde [97].

Ethanol accelerates yeast chronological aging via two different mechanisms. Both of these mechanisms are elicited in response to ethanol-dependent suppression of the Fox1-, Fox2-, and Fox3-driven peroxisomal β-oxidation of fatty acids to acetyl-CoA [21,37]. Each of these two mechanisms is specific to the chronological age of a yeast cell.

In chronologically “young” yeast, the ethanol-dependent suppression of acetyl-CoA formation in peroxisomes decreases its availability for anaplerotic conversion to citrate and acetyl-carnitine in these organelles [3,21,37]. This accelerates yeast chronological aging by weakening the longevity assurance process of supplying citrate and acetyl-carnitine to mitochondria for the replenishment of these two TCA cycle intermediates (Figure 1K) [3,21,37].

In chronologically “old” yeast, the ethanol-dependent suppression of peroxisomal β-oxidation of fatty acids leads to the excessive accumulation of free (non-esterified) fatty acids (FFA) [3,21,37]. Such build-up of FFA accelerates yeast chronological aging by increasing the risk of an age-related mode of regulated cell death (RCD) called “liponecrosis” (Figure 1K) [3,98,99,100].

### 2.11. FFA and Diacylglycerol (DAG)

The ethanol-driven excessive accumulation of unoxidized FFA in yeast peroxisomes (see Section 2.10) elicits several negative-feedback loops whose action causes a buildup of FFA and DAG in the ER and lipid droplets (LD) [3,100,101]. This buildup of FFA and DAG accelerates the onset of the liponecrotic mode of RCD, thereby increasing the risk of death and accelerating yeast chronological aging (Figure 1L) [3,21,98,99,100]. Thus, both FFA and DAG are pro-aging metabolites that shorten yeast CLS.

## 3. The Spatiotemporal Dynamics of Changes in Concentrations of Some Metabolites Define Yeast CLS

It is becoming increasingly evident that the concentrations of the key metabolites that influence the pace of yeast chronological aging are controlled spatially and temporally at different levels.

One level of the spatial control over the concentrations of these metabolites consists in regulating their intracellular and extracellular concentrations [3,102]. Some of the key metabolites can influence longevity-defining processes only within the cell they were produced; these metabolites: (1) define the pace of yeast chronological aging only via cell-autonomous mechanisms; (2) cannot act as low molecular weight transmissible longevity factors; and (3) include NADPH, trehalose, sphingolipids, FFA, and DAG [3,24,28,32,35,37,41,102]. In contrast, some of the key metabolites can influence longevity-defining processes both within the cell they were generated as well as within other cells in the yeast population; these metabolites: (1) define the pace of yeast chronological aging via both cell-autonomous and cell-non-autonomous mechanisms; (2) can act as low molecular weight transmissible longevity factors; and (3) include glycerol, H_2_O_2_, amino acids, spermidine, H_2_S, acetic acid, and ethanol [3,15,36,50,51,52,54,71,72,73,82,85,93,94,97,102]. It is noteworthy that some of those metabolites that cannot act as low molecular weight transmissible longevity factors (and thus define the pace of yeast chronological aging only via cell-autonomous mechanisms) may change metabolism within the “host” cell so that this cell may respond by altering the production of acetic acid and/or other metabolites that can act via both cell-autonomous and cell-non-autonomous mechanisms.

Another level at which the concentrations of these key metabolites are controlled spatially consists in regulating their abundance in different organellar compartments within the cell. The effective concentrations of the metabolites that influence the pace of yeast chronological aging are established and maintained within an intricate network that involves the unidirectional and/or bidirectional flow of certain metabolites between mitochondria, the nucleus, vacuoles, peroxisomes, the ER, the plasma membrane, LD, and the cytosol [22,23,27].

In addition, the concentrations of the key metabolites that define yeast CLS are controlled temporally. Indeed, it has been found that: (1) these metabolite concentrations undergo age-related changes; and (2) the effects of these metabolites on their protein sensors are also subjected to age-related changes; these protein sensors respond to concentration changes of the key metabolites by altering the efficiencies of certain longevity-defining cellular processes [3,27]. It has been therefore proposed that the concentration changes of different key metabolites are temporally limited to certain longevity-defining periods called “checkpoints” [2,3,27,41,103]. Many of these checkpoints exist early in the life of chronologically aging yeast cultured in glucose-containing liquid media, during diauxic and post-diauxic growth phases [3,27]. NADPH, glycerol, H_2_O_2_, amino acids, sphingolipids, and spermidine are the key metabolites whose concentrations only at these “early” checkpoints play essential roles in defining the pace of yeast chronological aging [24,27,28,32,35,36,42,51,52,53,54,71,72,73,82]. Some of these checkpoints are the “late” checkpoints that occur after chronologically aging yeast cultured in glucose-containing liquid media undergoes cell cycle arrest and enters the non-proliferative stationary phase of culturing [3,27]. H_2_S, acetic acid, FFA, and DAG are the key metabolites whose concentrations only at these “late” checkpoints make essential contributions to defining the pace of yeast chronological aging [15,27,37,85,93,94]. It is noteworthy that trehalose and ethanol are the two key metabolites whose concentrations affect the longevity of chronologically aging yeast at both the “early” and the “late” checkpoints; for each of these two metabolites, the longevity-defining effect at the “early” checkpoint differs from that at the “late” checkpoint (see Section 2.3 and Section 2.10) [3,27,41]. It has been also proposed that at each of these “early” and “late” checkpoints, the concentration changes of the key metabolites are detected by a distinct set of checkpoint-specific protein sensors. These protein sensors have been called “master regulators” because their suggested common role consists in responding to the concentration changes of different key metabolites by altering the rates and efficiencies of various longevity-defining cellular processes throughout the chronological lifespan, thus establishing a pro- or anti-aging cellular pattern and defining yeast CLS [3,27].

## 4. Conclusions

Based on our analysis of how the spatiotemporal dynamics of changes in the concentrations of some metabolites influence yeast CLS, we conclude that a distinct group of metabolites can act as second messengers that define the pace of yeast chronological aging. Molecules that can operate both as intermediates of yeast metabolism and as second messengers of yeast chronological aging include NADPH, glycerol, trehalose, H_2_O_2_, amino acids, sphingolipids, spermidine, H_2_S, acetic acid, ethanol, FFA, and DAG. Akin to second messengers of signal transduction [104,105], these second messengers of yeast chronological aging: (1) are generated and can undergo substantial concentration changes within a yeast cell in response to some extracellular stimuli (i.e., nutrients and hormetic stresses); these extracellular stimuli are detected by certain hierarchically organized proteins and protein complexes (i.e., the TORC1 and PKA signaling pathways) that then transduce the signal to some metabolic reactions involved in the generation of the intracellular second messengers; (2) differ from each other in their chemical properties and solubilities; these differences allow the second messengers to transduce the signal in the polar chemical environment of the cytosol or organelle interior, be distributed within the hydrophobic chemical environment of the cellular or organellar membrane, or be translocated across such membranes as a gas or free radical; (3) are subjected to stringent spatial control; indeed, the movement of these second messengers within the cell and their exit out of the cell are regulated in response to changes in the nutrient, energy, stress, and/or proliferation status of the cell; (4) are also controlled temporally, as their intracellular and extracellular concentrations at different periods of chronological lifespan vary significantly and are regulated by alterations in nutrient availability, stress intensity, and cell proliferation rate; and (5) are detected by a distinct set of ligand-specific protein sensors that respond to the concentration changes of different second messengers by altering the rates and efficiencies of longevity-defining cellular processes, thereby creating a pro- or anti-aging cellular pattern and affecting the pace of yeast chronological aging.

Of note, Pietrocola et al. [26] have recently concluded that acetyl-CoA can operate both as a metabolic intermediate and as a second messenger in regulating the delicate balance between cellular catabolism and anabolism in eukaryotic organisms across phyla. In conjunction with the demonstration that acetyl-CoA plays essential roles in regulating the longevity of chronologically aging yeast (see Section 2.9 and Section 2.10; [21,25,37,95,96]), this important conclusion further supports the notion that intermediates of some metabolic pathways in eukaryotic cells can function as second messengers that orchestrate global changes in cell functionality in response to changes in the nutrient, energy, stress, and proliferation status of the cell.

## Abbreviations

Asp	aspartate
Asn	asparagine
ATG	autophagy-related genes
ER	endoplasmic reticulum
ETC	electron transport chain
FFA	free (non-esterified) fatty acids
CLS	chronological lifespan
CR	caloric restriction
DAG	diacylglycerol
DDR	DNA damage response
Glu	glutamate
Gln	glutamine
GTR	glutathione reductase
LD	lipid droplets
PKA	protein kinase A
Pkh1	Pkb-activating kinase homolog protein 1
Pkh2	Pkb-activating kinase homolog protein 2
RCD	regulated cell death
ROS	reactive oxygen species
TCA	tricarboxylic acid
TORC1	target of rapamycin complex 1
TRR	thioredoxin reductase
TSP	transsulfuration pathway
